# Development of a mono-promoter-driven CRISPR/Cas9 system in mammalian cells

**DOI:** 10.1038/srep18341

**Published:** 2015-12-16

**Authors:** Shin Yoshioka, Wataru Fujii, Tetsuhiro Ogawa, Koji Sugiura, Kunihiko Naito

**Affiliations:** 1 Department of Animal Resource Sciences, Graduate School of Agricultural and Life Sciences, The University of Tokyo, Yayoi 1-1-1, Tokyo 113-8657, Japan; 2 Department of Biotechnology, Graduate School of Agricultural and Life Sciences, The University of Tokyo, Yayoi 1-1-1, Tokyo 113-8657, Japan

## Abstract

The CRISPR/Cas9 system has been used for spatio-temporal gene modification through the ubiquitous expression of gRNA by an RNA polymerase III promoter and the controlled expression of Cas9 using a tissue-specific or inducible promoter. However, unexpected gene disruptions indicate the necessity of a tissue-specific or inducible expression of not only Cas9 but also gRNA. In the present study, we attempted to develop a CRISPR/Cas9 system that could express functional gRNAs and Cas9 by a single RNA polymerase II promoter and induce multi-loci disruptions in specific cells. To this end, we designed vectors expressing ribozyme-flanked gRNAs (RGRs) and Cas9 mRNAs simultaneously. We showed that the mono-promoter-driven vector induces gene disruptions at the target loci in HEK 293 cells after transfection. In addition, two target loci were disrupted simultaneously by the transfection of a mono-promoter-driven vector expressing two RGRs and Cas9 mRNA. Finally, we constructed a universal vector for use in the construction of plasmids to be applied to the present mono-promoter-driven CRISPR/Cas9 system. We have thus provided a versatile tool for generating gene disruptions by the CRISPR/Cas9 system; this system should contribute to a wide range of investigations, including studies on spatio-temporal gene functions.

Conditional knockout (KO) animals, in which targeted disruptions can be induced artificially or regulated spatio-temporally, enable the elucidation of gene functions in specific organs or cell types at various time points, even for embryonic lethal genes^1^. Although the spatio-temporal disruption of a target gene has been achieved by using the Cre-loxP system in mice, this method requires flanking the target gene by loxP sequences in embryonic stem (ES) cells through homologous recombination, injection of targeted ES cells into blastocysts to obtain germline chimeric animals, generation of targeted-gene-floxed animals and mating with animals expressing the Cre-recombinase under the cell-type specific promoter^2^. Thus, the generation of conditional KO mice using the Cre-loxP system is painstaking, time-consuming and expensive.

The CRISPR/Cas9 system, which consists of Cas9 endonuclease and guide-RNA (gRNA), can induce site-specific DNA double-strand breaks and resulting target mutations, and it has been developed as a genome modification tool in diverse cell types and organisms^3^,^4^,^5^,^6^,^7^. The CRISPR/Cas9 system is expected to be utilized for spatio-temporal gene modification. In the generally used CRISPR/Cas9 system, RNA polymerase III promoters (e.g., U6 promoter and H1 promoter) are used for the transcription of gRNA because the RNA polymerase II promoter adds extra nucleotides to the 5′- and 3′-ends of gRNA and interrupts the normal gRNA function. In contrast, RNA polymerase III promoters produce the transcripts without any additional nucleotides but work ubiquitously^8^ and cannot be used for the spatio-temporal control of gRNA expression. In the case of Cas9, the mRNA can be transcribed by RNA polymerase II promoters^4^, and therefore, a tissue-specific promoter can be used for the Cas9-expressing vectors just as for the Cre-expressing vectors^9^,^10^. At present, animals with conditional genome modifications generated by the CRISPR/Cas9 system have been reported in *Caenorhabditis elegans* and *Drosophila*, and these modifications are controlled only by the specificity of Cas9 mRNA expression^9^,^10^,^11^. However, in a study on *Drosophila*, gRNA expressed by the U6 promoter and Cas9 expressed by the vasa promoter, which were expected to lead to the germ-line–specific expression of Cas9, introduced gene disruption not only in the germ line but also at unexpected sites^10^. In mammalian cells, a recent study noted the possibility of off-doxycycline mutations in the continuous expression of gRNA with the regulatory expression of Cas9 by a Tet-on system *in vivo* and *in vitro*^12^. In addition, care should be taken when adopting this technique in the future, as the ubiquitous and continuous gRNA expression in various extraneous tissues might have some deleterious effects on the phenotypes of the animals. Therefore, the regulatory expression of both gRNA and Cas9 should be suitable to achieve a fully controllable spatio-temporal gene modification rather than only Cas9 regulation.

Hammerhead (HH) ribozyme^13^ and hepatitis delta virus (HDV) ribozyme^14^ perform site-specific self-cleavage, resulting in cleavage products with 2′,3′-cyclic phosphate and 5′-hydroxyl termini, respectively^15^. Recently, HH and HDV ribozymes were cloned into an expression plasmid at the at the 5′-end and 3′-end of a defined sgRNA sequence to process the sgRNA transcribed by RNA polymerase II in yeast and mammalian cells^16^,^17^. This ribozyme-flanked gRNA (RGR) expression system is expected to be used for the spatio-temporal expression of gRNA by tissue-specific promoters.

In the present study, we established a mono-promoter-driven CRISPR/Cas9 system expressing both gRNA and Cas9 by using an RNA polymerase II promoter and examined whether this mono-promoter-driven CRISPR/Cas9 system could be used for genome modification in mammalian cells. The mono-promoter-driven method may also contribute to resolve problems such as relatively large molecular size of the cell type-specific promoters and the difficulty of generating gRNA and Cas9 double-transgenic animals. Finally, we assessed whether the mono-promoter-driven CRISPR/Cas9 system could generate a multiple-gene disruption.

## Results

### Expression of a functional gRNA by an RNA polymerase II promoter in a human cell line

**Figure 1:**
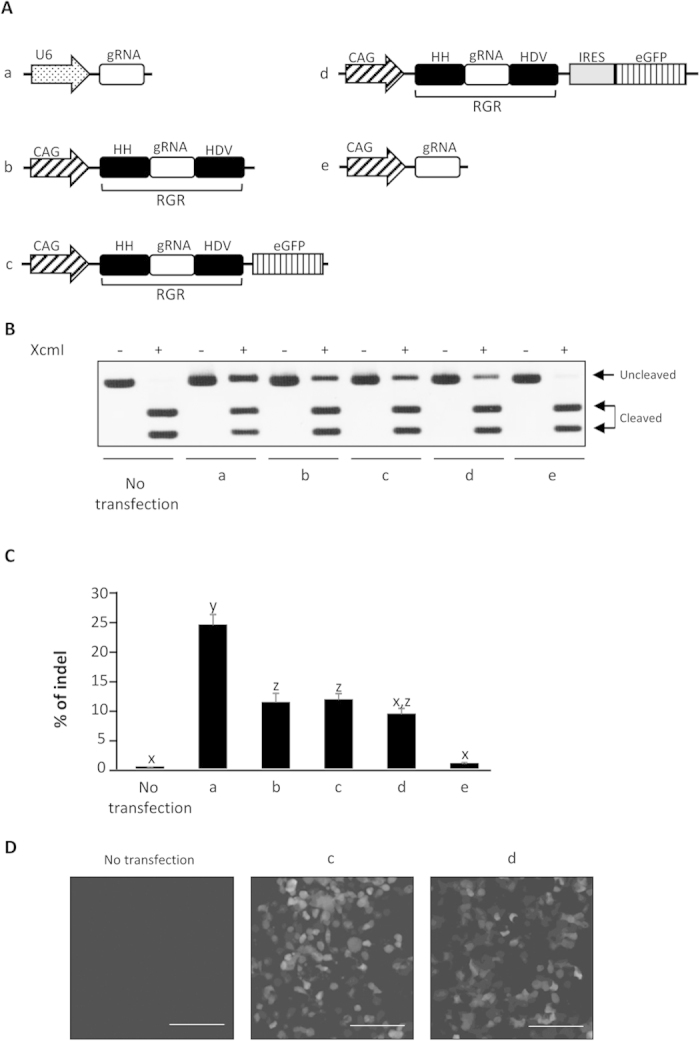
Expression of functional gRNAs by a RNA polymerase II promoter in a human cell line. (**A**) Schematics of the gRNA expression vectors tested in (**B**–**D**). All gRNAs targeted *HPRT1*. (**B**) Restriction fragment-length polymorphism analysis. HEK 293 cells (1 × 10^5^) were co-transfected with pCAG-Cas9 (500 ng) and one of the vectors (500 ng) shown in (**A**), and the genomic DNAs of the cells were extracted 72 h after transfection. The DNAs were subjected to PCR, and 300 ng of the PCR products were subjected to agarose gel electrophoresis after incubation with or without XcmI. Cleaved and uncleaved fragments indicate unmodified and modified genomes, respectively. (**C**) The indel rates of the human *HPRT1* locus in HEK 293 cells were calculated from RFLP analysis, as shown in B. The band intensity was analyzed by ImageJ software. All values represent means ± SEM of three separate experiments. Different letters indicate significant differences (P < 0.05), as determined by ANOVA followed by Tukey’s multiple comparison test (n = 3). (**D**) Representative images of HEK 293 cells 72 h after transfection with gRNA and eGFP expressing vectors. The scale bars represent 100 μm.

To investigate whether the single RNA polymerase II promoter could achieve the simultaneous expression of a functional gRNA and protein-coding mRNA, we examined the mutation-induction ability of gRNAs and the expression of the reporter protein using plasmids encoding *HPRT1*-targeted RGR connected with eGFP directly or via IRES (Fig. 1A). The mutation-induction ability of gRNA was evaluated after pCAG-Cas9 co-expression in the cells. The RFLP analysis and direct sequencing revealed that all of the RGR-type gRNA induce target mutations as the U6-gRNA by Cas9 co-expression (Fig. 1B and Supplementary Figure 1). The indel rates of RGR-type gRNAs were significantly lower than that of U6-gRNA, reflecting the lower expression of gRNA (Supplementary Figure 2), but there were no significant differences in rate among pCAG-RGR, pCAG-RGR-eGFP and pCAG-RGR-IRES-eGFP (Fig. 1C). The off-target mutation signal of these RGR-type gRNAs as well as U6-gRNA were examined at two potential off-target sites, and no off-target mutation signal was found at either site (Supplementary Figure 3). Conversely, pCAG-gRNA, which expresses ribozyme-free gRNA by RNA polymerase II, induces mutations modestly, indicating that gRNA processing by the ribozyme is indispensable for the expression of functional gRNA by RNA polymerase II (Fig. 1B,C). The eGFP expression was observed in both pCAG-RGR-eGFP- and pCAG-RGR-IRES-eGFP-transfected cells (Fig. 1D and Supplementary Figure 4). These results indicate the expression of both functional gRNA and protein by the single CAG promoter.

### Effectiveness of the mono-promoter-driven CRISPR/Cas9 system in a human cell line

**Figure 2:**
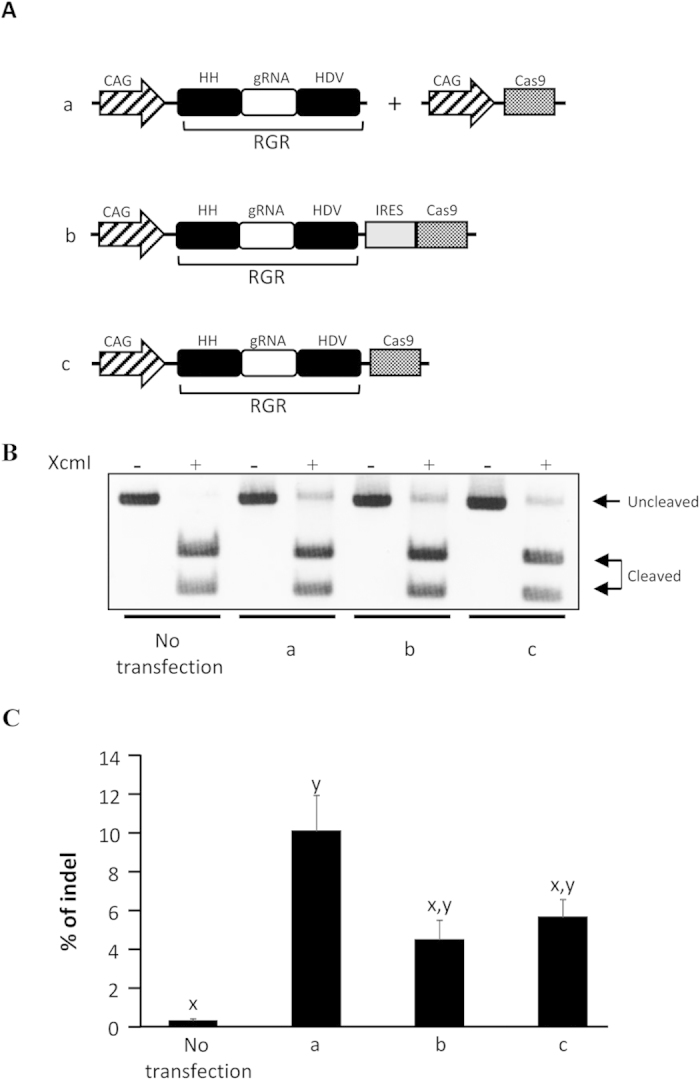
Effectiveness of the mono-promoter-driven CRISPR/Cas9 system in a human cell line. (**A**) Schematics of the gRNA and Cas9 expression vectors tested in (**B**,**C**). All gRNAs targeted *HPRT1*. (**B**) Restriction fragment-length polymorphism analysis. HEK 293 cells (1 × 10^5^) were transfected with one of the vectors (1 μg)/vector sets (500 ng each) shown in (**A**), and the genomic DNAs of the cells were extracted 72 h after transfection. The DNA was subjected to PCR, and 300 ng of the PCR products was subjected to agarose gel electrophoresis after incubation with or without XcmI. Cleaved and uncleaved fragments indicate unmodified and modified genomes, respectively. (**C**) The indel rates of the human *HPRT1* locus in HEK 293 cells were calculated from RFLP analysis, as shown in (**B**). The band intensity was analyzed by ImageJ software. All values represent means ± SEM of three separate experiments. Different letters indicate significant differences (P < 0.05), as determined by ANOVA followed by Tukey’s multiple comparison test (n = 3).

We prepared two plasmids, pCAG-RGR-IRES-Cas9 and pCAG-RGR-Cas9, which encode RGR and Cas9 with and without IRES mediation, respectively (Fig. 2A). Then, we examined whether these mono-promoter-driven CRISPR/Cas9 systems enable genome modification. The RFLP analysis and direct sequencing revealed that both plasmids induced the target mutations (Fig. 2B and Supplementary Figure 1). A comparison of the indel rates reveals that there is no significant difference between the individually driven group and the mono-promoter-driven groups, although the rate tended to be higher in the individually driven system than in the mono-promoter-driven-systems (Fig. 2C), as expected from its higher expression of Cas9 protein (Supplementary Figure 5C). Almost the same indel levels were obtained for pCAG-RGR-IRES-Cas9 and pCAG-RGR-Cas9 (Fig. 2C). The off-target mutation signal was not detected in these mono-promoter-driven systems (Supplementary Figure 3). We also obtained results indicating that the present mono-promoter-driven CRISPR/Cas9 system is effective for the doxycycline-inducible expression of CRISPR/Cas9-mediated genome modification in mammalian cells (Supplementary Figure 6).

### Multi-loci disruption using the mono-promoter-driven CRISPR/Cas9 system

**Figure 3:**
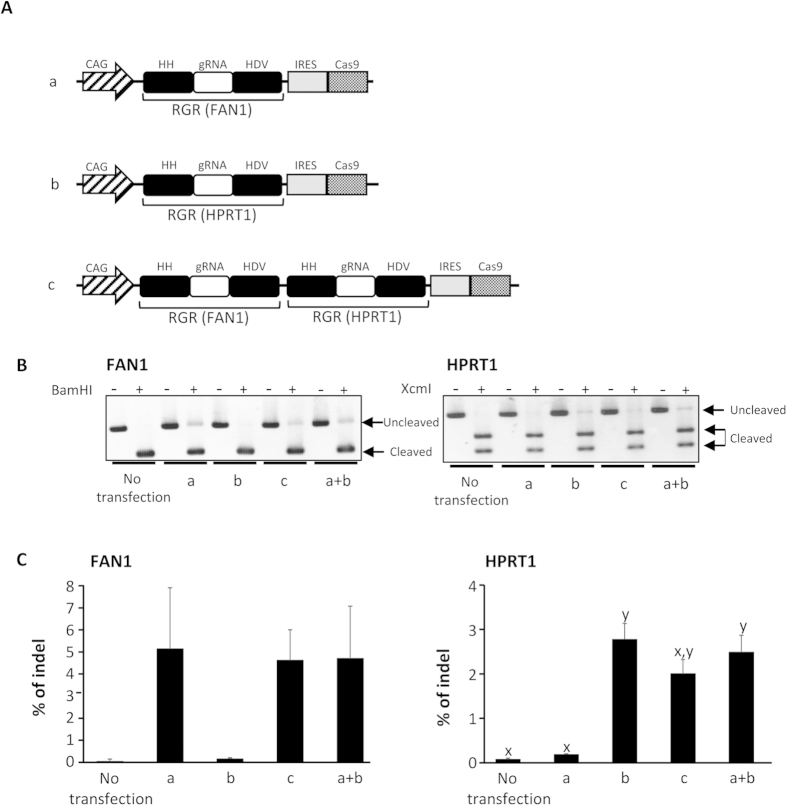
Multi-gene disruption using the mono-promoter-driven CRISPR/Cas9 system. (**A**) Schematics of the gRNA and Cas9 expression vectors tested in (**B**,**C**). All gRNAs targeted *HPRT1* and/or *FAN1*. (**B**) Restriction fragment-length polymorphism analysis. HEK 293 cells (1 × 10^5^) were transfected with one of the vectors (1 μg) shown in (**A**), and the genomic DNAs of the cells were extracted 72 h after transfection. The DNA was subjected to PCR, and 300 ng of the PCR products were subjected to agarose gel electrophoresis after incubation with or without XcmI for *HPRT1* and BamHI for *FAN1*. Cleaved and uncleaved fragments indicate unmodified and modified genomes, respectively. (**C**) The indel rates of the human *HPRT1* and *FAN1* loci in HEK 293 cells were calculated from RFLP analysis, as shown in (**B**). The band intensity was analyzed by ImageJ software. All values represent means ± SEM of three separate experiments. Different letters indicate significant differences (P < 0.05), as determined by ANOVA followed by Tukey’s multiple comparison test (n = 3).

To investigate whether the present mono-promoter-driven CRISPR/Cas9 system is applicable for multi-loci disruption, we constructed a mono-promoter-driven plasmid containing tandem-duplicated RGR targeting *HPRT1* and *FAN1* in addition to Cas9 (Fig. 3A). The RFLP analysis reveals that the *HPRT1* and *FAN1* loci were disrupted simultaneously by this multi-loci mono-promoter-driven plasmid in the same manner as by the single-locus mono-promoter-driven plasmids (Fig. 3B and Supplementary Figure 1). The indel rates induced by the single-locus target plasmids and multi-loci target plasmid are not significantly different (Fig. 3C), reflecting the comparable CAS9 protein expression amounts and the possibly similar gRNA expression amounts for each target between the single-locus target plasmids and multi-loci target plasmid (Supplementary Figures 2 and 5). These results suggest that the present mono-promoter-driven CRISPR/Cas9 system would be applicable for the disruption of multiple loci.

### Construction of a universal vector

**Figure 4:**
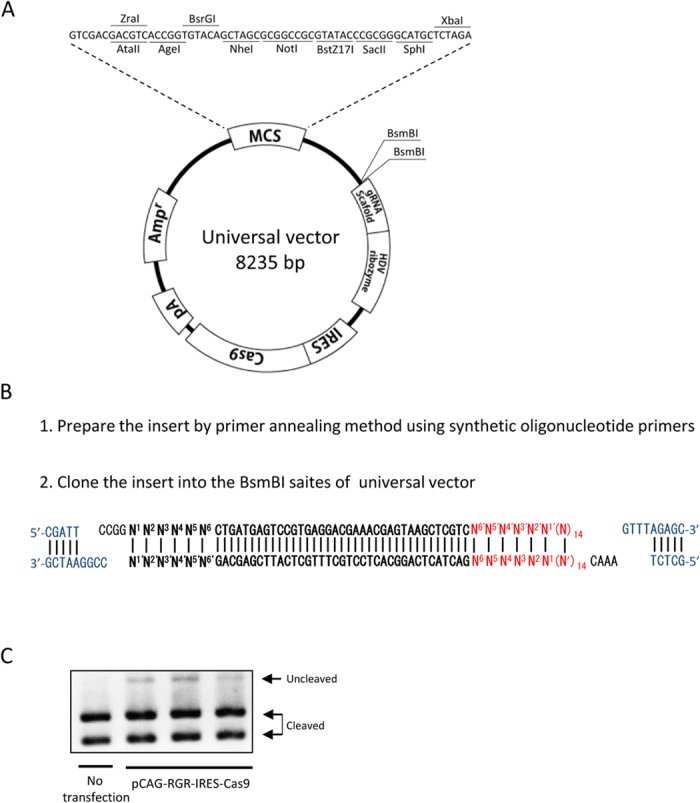
Universal vector for mono-promoter-driven CRISPR/Cas9 system. (**A**) Schematic illustration of the universal vector. (**B**) Sequence of RGR insertion. The portion of the RGR sequence containing additional nucleobases, which is necessary for the ligation, is prepared by the annealing of single strand oligonucleotides, followed by insertion into the BsmBI-digestion site of the plasmid vector. Blue characters indicate the sequence of the universal vector, red characters indicate the target sequence and bold characters indicate the HH ribozyme sequence. The first 6 bases of the HH ribozyme, from N^1^ to N^6^, must be matched with the 5′-end of the gRNA (underlined) for the function of HH ribozyme. (**C**) Restriction fragment-length polymorphism analysis. HEK 293 cells (1 × 10^5^) were transfected with *HPRT1* targeting pCAG-RGR-IRES-Cas9 vector (1 μg) reconstructed from the universal vector, and the genomic DNA of the cells was extracted 72 h after transfection. The DNA was subjected to PCR, and 300 ng of the PCR products was subjected to agarose gel electrophoresis after incubation with XcmI. Experiments were conducted using 3 plasmid clones of the same vector, and the results were similar to those of non-transfected cells. Cleaved and uncleaved fragments indicate unmodified and modified genomes, respectively.

To facilitate the construction of a mono-promoter-driven CRISPR/Cas9 plasmid with tissue-specific or inducible promoters and locus-specific gRNAs, a universal vector was prepared (Fig. 4A). Any promoters could be inserted into the restriction enzyme site of the multi-cloning sequence (Fig. 4A). A portion of the RGR sequence consisting of an HH ribozyme sequence, a target recognition sequence of gRNA and a 4-base overhang at both 3′ ends (Fig. 4B) must be synthesized by the annealing of single strand oligonucleotides, and then this sequence can be inserted into the double BsmBI sites. The first 6 bases of the HH ribozyme, from N^1^ to N^6^, must be matched with the 5′-end of the gRNA (underlined). In the present study, we reconstructed the *HPRT1*-targeting pCAG-RGR-IRES-Cas9 vector from the universal vector and confirmed that the pCAG-RGR-IRES-Cas9 derived from the universal vector could induce gene disruption (Fig. 4C). Thus, this plasmid enables the construction of the mono-promoter-driven CRISPR/Cas9 plasmid by 2 sub-cloning steps. The universal plasmid was deposited to Addgene (#64668).

## Discussion

In the present study, we demonstrated that ribozyme-flanked gRNA and Cas9 expression by a single CAG promoter could introduce mutations into multiple target loci of HEK 293 cells. Only a modest production of functional gRNAs by RNA polymerase II promoters was detected in the absence of ribozymes, presumably by the addition of the 5′ Cap structure, indicating that a ribozyme-flanked gRNA (RGR) expression system that utilizes the self-cleavage activity of ribozymes is essential for the functional gRNA production by RNA polymerase II promoters. In addition, an IRES sequence, which is generally essential for the control of more than two genes by a single promoter, was dispensable in genome disruption by the present mono-promoter-driven CRISPR/Cas9 system. Because the 5′ Cap structure is important for the proper initiation of protein translation, and cleavage by HH/HDV ribozyme results in the removal of the 5′ Cap structure from the mRNA, the Cas9 protein could not be translated from the ribozyme-isolated Cas9 mRNA. Therefore, the genome disruption by the mono-promoter-driven system without IRES might be due to the production of CAS9 protein from non-processed and/or pre-processed transcripts at the translation-initiation site after the Kozak sequence. The relatively lower mutation rates in the mono-promoter-driven systems relative to that in the individually driven system might be due to their much lower Cas9 expression levels. However, the difference was not significant, and this supports the previous suggestion that the concentration of gRNA is the main rate-limiting factor for target modification in a CRISPR/Cas9 system^7^, although improvement in the Cas9 expression is obviously also necessary.

Multiple-gene mutation is an effective research technique in various fields requiring multiple-gene-targeting, such as family genes, regulons, and quantitative traits. The CRISPR/Cas9 system has been applied in these fields, resulting in the successful generation of multiple-gene modifications in several animals and plants^6^,^10^,^18^,^19^,^20^,^21^,^22^. In the present study, we demonstrated that the *HPRT1* and *FAN1* loci could be simultaneously disrupted by the mono-promoter-driven CRISPR/Cas9 system. In addition, the efficiencies of mutagenesis were not significantly different between the multiple-gene mutation and two single-gene mutations. Thus, this system would be useful for generating multiple mutants affecting two or even more target loci using only one plasmid vector. The present mono-promoter-driven CRISPR/Cas9 system is expected to be effective for applications requiring the large-scale deletion of a target genome locus^3^,^7^ or the offset-nicking method^23^,^24^, which need multiple gRNAs.

Although a ribozyme-flanked gRNA (RGR) expressed by RNA polymerase II promoter could induce the targeted gene disruption in the present study, the indel rates were significantly lower than those in the conventional CRISPR/Cas9 system expressing gRNAs under a U6 promoter. Because a previous study suggested that the concentration of gRNA is the rate-limiting factor for target modification in a CRISPR/Cas9 system^7^, as described above, and the CAG promoter is known to efficiently transcribe DNA into RNA, the self-cleavage ability of the ribozymes used in the present study might be insufficient. The amount of gRNA produced from each RGR of multiple RGRs might be comparable with that from a single RGR, so one solution to this problem would be the tandem multiplication of RGRs. Evaluating other self-cleavage systems, such as Csy4-mediated processing^17^ or the tRNA-processing system^25^, might also be valuable for the improvement of the mono-promoter-driven CRISPR/Cas9 system.

We constructed a universal vector for the mono-promoter-driven CRISPR/Cas9 system. This vector contains a multi-cloning site for the addition of the desired promoter and double BsmBI sites for the addition of the desired gRNA, as well as the Cas9-coding sequence (Fig. 4). This universal vector is expected to assist in future spatio-temporal gene modifications using the CRISPR/Cas9 system.

Recent studies have reported on spatio-temporal gene modifications using the CRISPR/Cas9 system, which expresses gRNA ubiquitously under the control of a U6 promoter and Cas9 under the control of a tissue-specific or inducible promoter. However because unexpected gene disruptions were also observed in these studies^11^,^12^, there is a need for further improvement to the tissue-specificity or inducibility of the promoters. These unexpected gene disruptions were likely due to ubiquitous gRNA expression and leaky Cas9 expression. We demonstrated that ribozyme-flanked gRNA and Cas9 expression by a single CAG promoter could introduce mutations into multiple target loci of HEK 293 cells. The CAG promoter used in the present system is an RNA polymerase II promoter and could be replaced by other RNA polymerase II-recognized tissue-specific or inducible promoters. In addition, the mono-promoter-driven CRISPR/Cas9 system leads to a smaller plasmid size than the conventional CRISPR/Cas9 system, which uses two different promoters for the gRNA and Cas9 expressions. This means that this system should achieve a more efficient generation of conditional knockout animals, and particularly the bacterial artificial chromosome (BAC)-mediated conditional expression because the large plasmid size may decrease the efficiency of transgenesis^26^. The present CRISPR/Cas9 system might help decrease both the risk of unexpected gene disruption and the deleterious effects of ubiquitous and continuous gRNA expression in various extraneous tissues of the treated animals, and thus it would be suitable for spatio-temporal genome modification. Further studies are clearly needed to determine whether this system can be used to generate conditional knockout animals.

## Materials and Methods

### Plasmid construction

Target sequences for human *HPRT1* were designed in the first exon of the human *HPRT1* gene, and those for *FAN1* were designed according to a previous work^27^ (Supplementary Table1). The sequences for the hammer head (HH) ribozyme and hepatitis delta virus (HDV) ribozyme were obtained from previous works^14^,^16^. The RGRs were synthesized by overlap-extension PCR using synthetic oligonucleotide primers (Supplementary Table 1). The PCR amplicon was inserted into the EcoRI site of the pCAGGS vector, and the resulting vector was referred to as pCAG-RGR. Enhanced green fluorescent protein (eGFP) with or without an internal ribosome entry site (IRES) was inserted into the BglII restriction site of pCAG-RGR, resulting in pCAG-RGR-eGFP or pCAG-RGR-IRES-eGFP, respectively. The Cas9-expressing vector (pCAG-Cas9) was constructed in a previous work^7^. The Cas9 ORF sequence of pCAG-Cas9 was inserted into the Acc65I restriction site of the pCAG-RGR-IRES-eGFP vector, yielding pCAG-RGR-IRES-Cas9. For the construction of the tandem RGR vector (pCAG-RGR-RGR-IRES-Cas9), the RGR was obtained from pCAG-RGR-IRES-Cas9 by digesting with XbaI and SpeI and then inserted into the XbaI restriction site of the pCAG-RGR-IRES-Cas9 vector. For construction of the universal vector, a sequence that contains double BsmBI sites, a gRNA scaffold and an HDV ribozyme was synthesized by overlap-extension PCR using synthetic oligonucleotide primers (Supplementary Table2), and the RGR region of the pCAG-RGR-IRES-Cas9 vector was replaced with the sequence. Then, the CAG promoter of the vector was replaced by a multiple cloning site (MCS), which was synthesized by annealing the oligonucleotide shown in Supplementary Table 2. The constructed vectors were sequenced using a commercial sequencing kit (Applied Biosystems, Foster City, CA, USA) and a DNA sequencer (Applied Biosystems) according to the manufacturer’s instructions. The sequences of these vectors are shown in Supplementary Figure 7.

### Cell culture and transfection

HEK 293 cells (1 × 10^5^) were seeded in each well of a 24-well poly-L-lysine (Sigma) coated plate, and cultured in Dulbecco’s modified Eagle’s medium (DMEM) supplemented with 10% FBS at 37 °C with 5% CO_2_. Before transfection, the medium was replaced by Opti-MEM (Life Technologies), and then a total of 1 μg of plasmid DNA was transfected by Lipofectamine LTX reagent (Life Technologies), according to the manufacturer’s instructions. Twenty-four hours later, the medium was replaced by DMEM supplemented with 10% FBS. The transfected cells were harvested 72 h after transfection using 0.25% trypsin-0.05% EDTA in PBS. The harvested cells were centrifuged, and the pellet was added with 300 μl of tail buffer (1% SDS, 0.1 M NaCl, 0.1 M EDTA and 0.05 M tris [pH8.0]) with 30 μl of proteinase K (20 mg/ml, TaKaRa) and incubated at 65 °C overnight. The genomic DNA was precipitated with ethanol, washed, and resuspended in DNase- and RNase-free water (GIBCO).

### Observation of eGFP expression

HEK293 cells (1 × 10^5^) were seeded on each chamber of a poly-L-lysine coated 4-chamber slide, and then 500 ng of eGFP-expressing vector was transfected into the HEK293 cells in a manner similar to that described above. Twenty-four, 48 and 72 hours later, the eGFP expression was observed by confocal microscopy (LSM 700, Carl Zeiss).

### Restriction fragment-length polymorphism (RFLP) analysis

PCR was performed using extracted DNAs with the primers shown in Supplementary Table 3 under the following conditions: 95 °C for 5 min; 35 cycles of 95 °C for 30 sec, 58 °C for 30 sec and 72 °C for 30 sec; 72 °C for 10 min and hold at 4 °C. The PCR amplicons were purified using a FastGene Gel/PCR extraction kit (NIPPON Genetics) following the manufacturer’s protocol. The PCR product (300 ng) was digested with XcmI for *HPRT1* or BamHI for *FAN1*, and the digested materials were measured by agarose-gel electrophoresis. The band intensity was analyzed by ImageJ software, and indel percentages were calculated according to a previous study with some modifications^3^.

### Statistical analysis

All experiments were repeated at least three times. The statistical significance of differences was assessed by analysis of variance (ANOVA) followed by Tukey’s multiple comparison test.

## Additional Information

**How to cite this article**: Yoshioka, S. *et al.* Development of a mono-promoter-driven CRISPR/Cas9 system in mammalian cells. *Sci. Rep.* **5**, 18341; doi: 10.1038/srep18341 (2015).

## Supplementary Material

Supplementary Information
