# Supplementary material for: Development of a mono-promoter-driven CRISPR/Cas9 system in mammalian cells
Source: Sci Rep. 2015 Dec 16;5:18341. doi: 10.1038/srep18341 (PMC4680873; doi:10.1038/srep18341)
Supplement: Supplementary Information [file srep18341-s1.pdf]

Supplementary Information

Development of a mono-promoter-driven CRISPR/Cas9 system in mammalian cells.

Shin Yoshioka, Wataru Fujii, Tetsuhiro Ogawa, Koji Sugiura, Kunihiko Naito

Supplementary Materials and Methods: (Page 2-3)

Supplementary Figure 1: Direct sequence analysis data. (Page 4)

Supplementary Figure 2: Comparison of gRNA expression level among the U6-gRNA, pCAG-RGR and mono-promoter types vectors. (Page 5)

Supplementary Figure 3: T7EI assay of potential off-target loci for *HPRT1*. (Page 6)

Supplementary Figure 4: The eGFP expression of pCAG-RGR-eGFP and pCAG-RGR-IRES-eGFP. (Page 7)

Supplementary Figure 5: Comparison of Cas9 expression level between the Cas9 individually-driven CRISPR/Cas9 system and mono-promoter-driven CRISPR/Cas9 system. (Page 8)

Supplementary Figure 6: Ubiquitous expression of Cas9 induce the off-doxycycline gene disruption. (Page 9)

Supplementary Figure 7: Sequence of vectors and RGR. (Page 10-14)

Supplementary Table 1: Target sequence of human *HPRT1* and *FAN1* locus and primer sequence for RGR. (Page 15)

Supplementary Table 2: Primer sequence for universal vector. (Page 16)

Supplementary Table 3: Primer sequence for RFLP analysis. (Page 17)

Supplementary Table 4: Primer sequence for RT-PCR. (Page 18)

Supplementary References: (Page 19)

Supplementary Materials and Methods

Direct sequence analysis

Uncleaved band from RFLP analysis was purified using FastGene Gel/PCR extraction kit (NIPPON Genetics) following manufacturer's protocol. The purified DNA was sequenced using commercial sequencing kit (Applied Biosystems, Foster City, CA, USA) and a DNA sequencer (Applied Biosystems) according to the manufacturer's instructions.

Immunocytochemistry

Cultured cells were fixed in 4% paraformaldehyde and permeabilized in 0.2% triton X-100. These cells were treated with an anti-Flag M2 monoclonal antibody (F1804, Sigma-Aldrich) overnight. After washing, the cells were incubated in fluorescein-isothiocyanate-conjugated anti-mouse IgG (55494, MP Biomedicals) for 60 min. DNA was visualized by 45 μg/ml propidium iodide in PBS for 30 min, and then observed under a confocal laser scanning microscope (LSM 700, Carl Zeiss).

T7 endonuclease I assay

Genomic DNA from transfected and no transfected HEK 293 cells was subjected to PCR using the primers shown in Supplementary Table 3. The PCR amplicons were purified by agarose-gel extraction following electrophoresis. T7 endonuclease I assay for PCR products was performed according to previous report [1].

4.

Western blot analysis

Western blot analysis was performed according to the previous report with some modifications [2]. The cells transfected with pCAG-Cas9 plus pCAG-RGR, pCAG-RGR-Cas9, pCAG-RGR-IRES-Cas9 and pCAG-RGR-RGR-IRES-Cas9 were suspended in Laemmli buffer [3]. The antibodies used were anti-Flag-M2 monoclonal antibody (1:500; F1840, Life technologies, USA) and anti-β-actin polyclonal antibody (1:3000; GTX109639, GeneTex, Irvune, CA, USA). To visualize the protein-bound antibodies, horseradish peroxidase-conjugated anti-mouse IgG and anti-rabbit IgG (Jackson ImmunoResearch Laboratories, West Grove, PA, USA) were used for the second layer, respectively, followed by a detection procedure using an ECL detection kit (Amersham-Pharmacia) according to the manufacturer’s protocol.

Northern blot analysis and RT-PCR

Total RNA for northern blot analysis and RT-PCR was extracted with TRIzol regent (Life Technologies) following manufacturer’s protocol. The extracted total RNA was quantified using Nanodrop (Thermo Scientific) and normalized to same concentration (200 ng/μl).

Northern blot analysis was carried out as previously described [4]. The DNA sequences for the specific probes were as follows: 5’-TTCAAGTTGATAACGGACTAGCCTT-3’ for gRNA and 5’-TTGAACCCTGGACCCTCAGA-3’ for $tRNA^{Lys}$ [5].

Total RNA was reverse-transcribed using ReverTra Ace (TOYOBO) according to the manufacturer’s protocol. Then, RT-PCR was carried out using TaKaRa Ex Taq with a GeneAtlas (ASTEC). The primer sets for RT-PCR were shown in Supplementary Table 4.

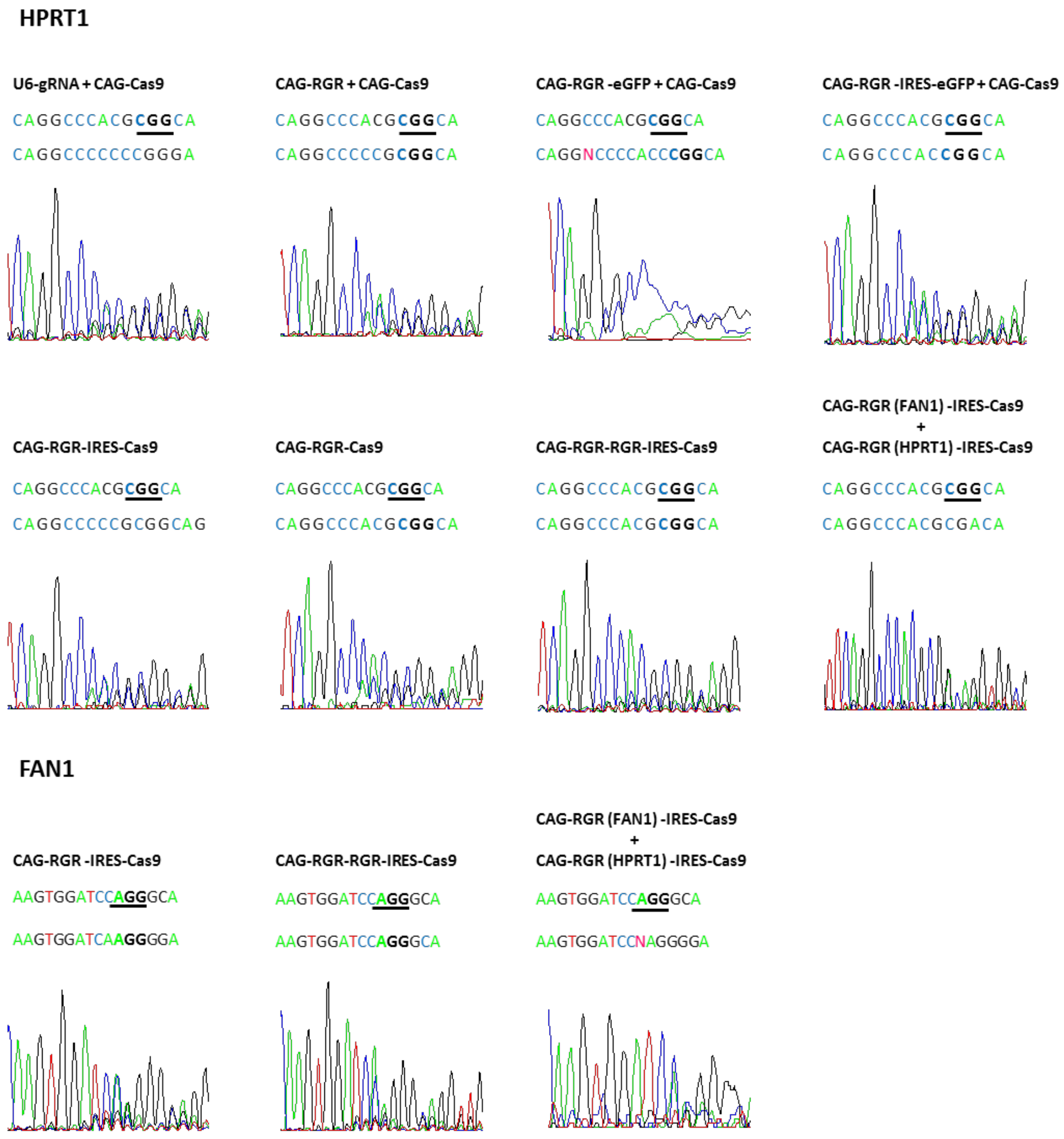

Supplementary Figure 1. Direct sequence analysis results. Representative image of the waveform data of a DNA sequencer obtained from an uncleaved band of RFLP analysis. Waveform is disarranged around the PAM domain. The upper nucleotides indicate the wild type sequences. The lower nucleotides indicate the sequences predicted from the waveform. The underline shows the PAM domain.

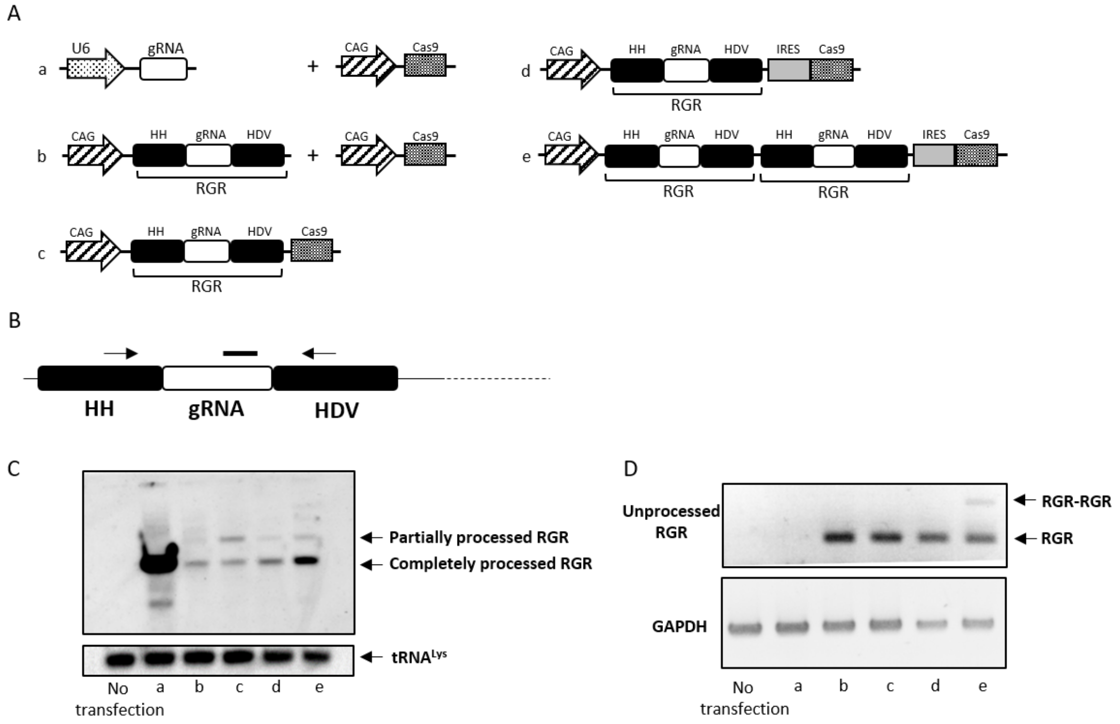

Supplementary Figure 2. Comparison of gRNA expression level among the U6-gRNA, pCAG-RGR and mono-promoter type vectors. (A) Schematics of the gRNA and Cas9 expression vectors tested in C and D. All gRNAs targeted *HPRT1* and/or *FAN1*. (B) Schematic illustration of RGR and site information of probe and primers used in this study. Line indicates probe for gRNA detection which designed within the gRNA scaffold. Arrows indicate the primers for unprocessed RGR detection which designed within the HH ribozyme (Forward primer) and the HDV ribozyme (Reverse primer). (C) Representative pictures of Northern blot analysis. HEK 293 cells (1×10^5) were transfected with one of the vectors (1 μg) and vector sets (500 ng each) shown in (A), and the total RNAs of the cells were extracted 72 h after transfection. The 2 μg of the total RNA was subjected to Northern blot analysis (n=3). Upper panel showed completely processed RGR and partially processed RGR. Lower panel showed $tRNA^{Lys}$ as an internal control. (D) Representative pictures of RT-PCR. The cDNA reverse transcribed from total RNA was subjected to RT-PCR. Upper panel showed unprocessed RGR and lower panel showed GAPDH as an internal control.

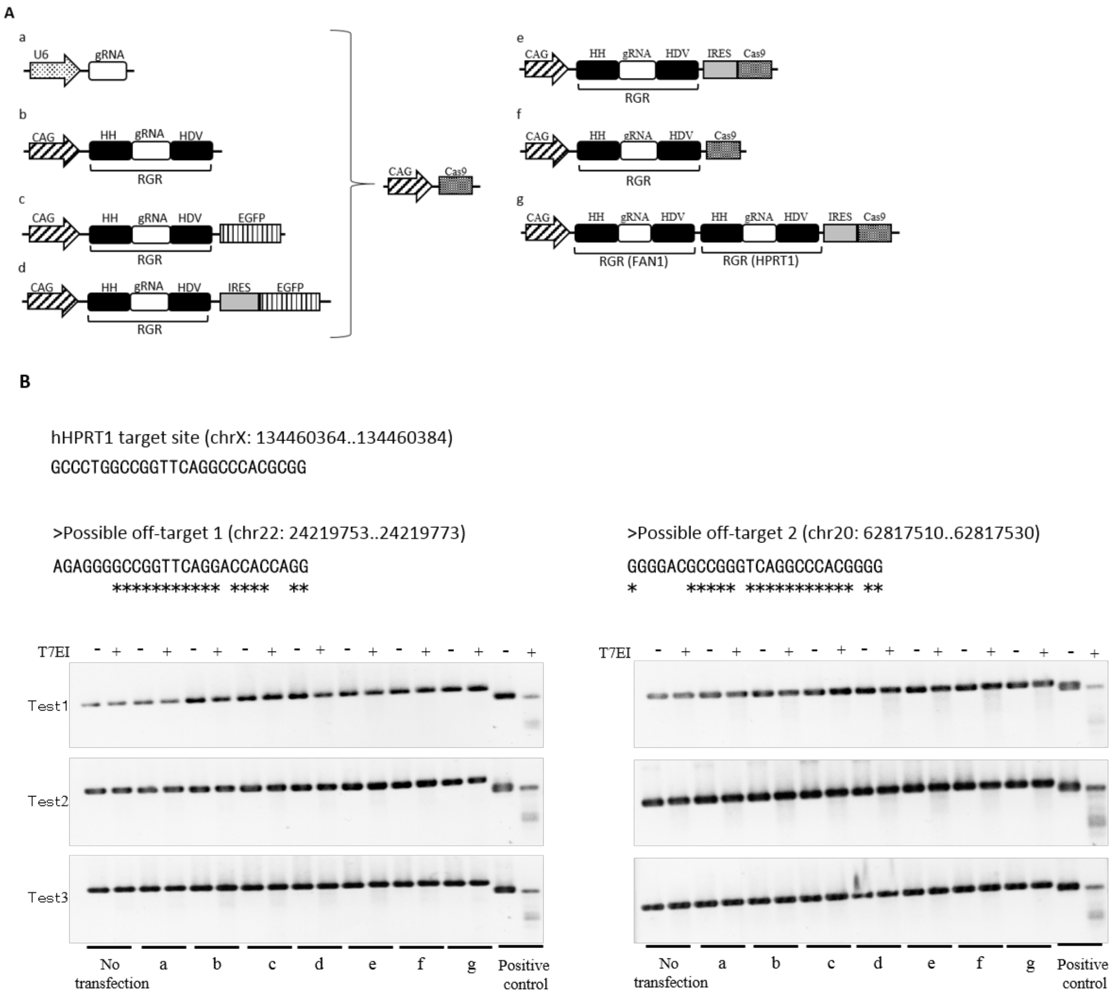

Supplementary Figure 3. T7EI assay of potential off-target loci for HPRT1. (A) Schematics of the gRNA expression vectors tested in B. All gRNAs targeted *HPRT1*. (B) HEK 293 cells ($1x10^5$) were transfected with one of the vectors (1 µg) and vector sets (500 ng each) and the genomic DNAs of the cells were extracted 72 h after transfection. The DNA was subjected to PCR and 250 ng of the PCR products were subjected to agarose-gel electrophoresis after incubation with or without T7 endonuclease I (n=3). The PCR amplicon derived from Rosa26 heterozygous mutated mouse was used as positive control for T7EI assay.

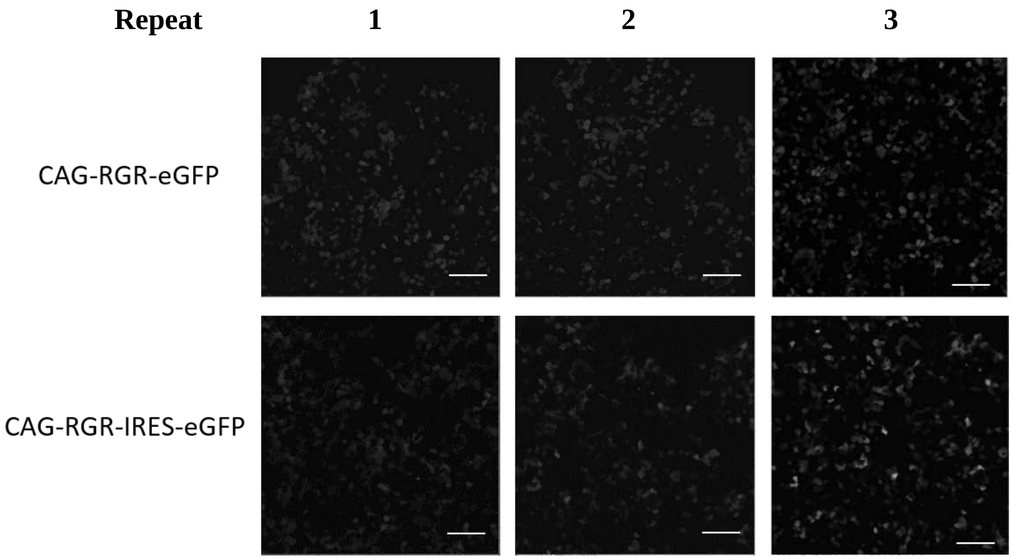

Supplementary Figure 4. The eGFP expression of pCAG-RGR-eGFP and pCAG-RGR-IRES-eGFP. Confocal microscopy observation of eGFP expression. HEK 293 cells ($1x10^5$) were transfected with pCAG-RGR-eGFP and pCAG-RGR-IRES-eGFP, and the eGFP expression was observed 72 h after transfection. We repeated three times and each result of three experiments are shown. The scale bars represent 100 μm.

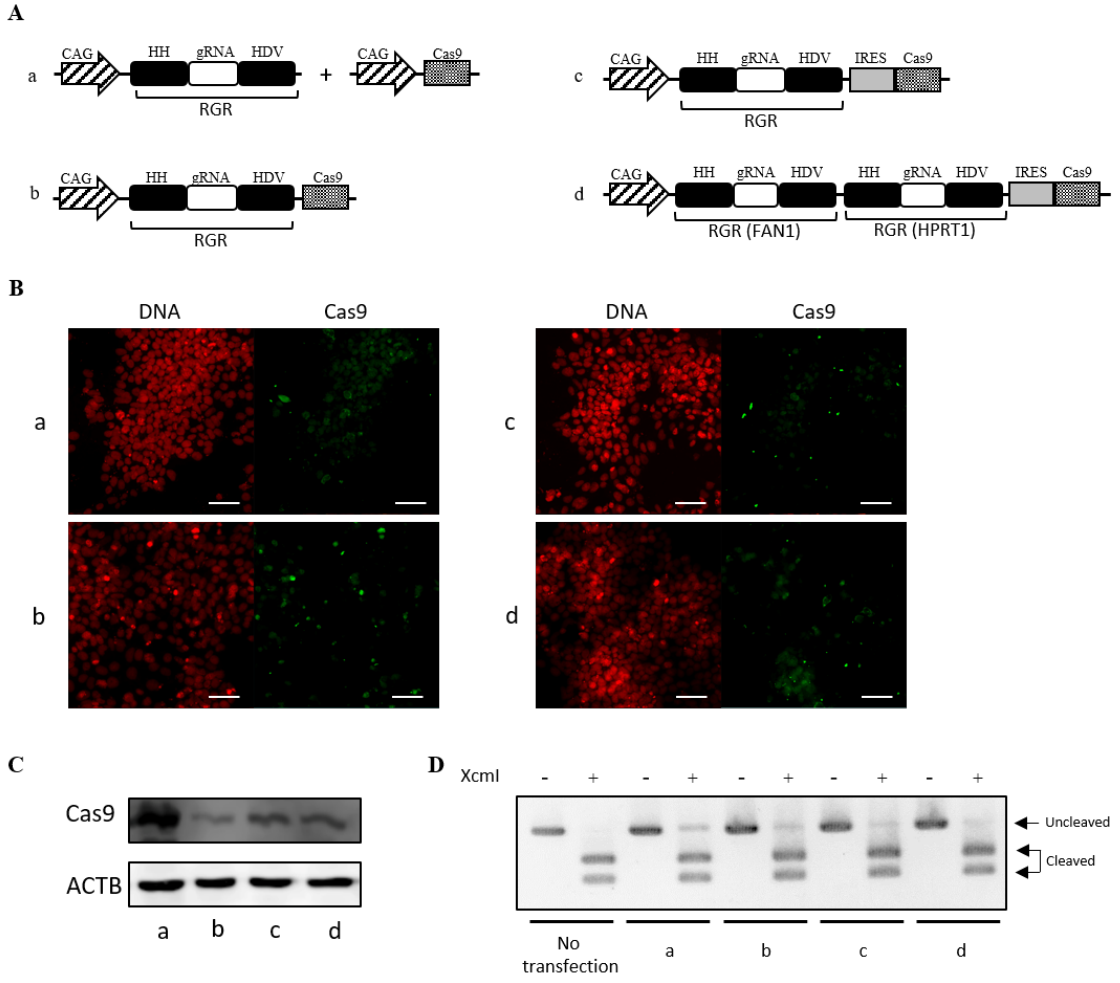

Supplementary Figure 5. Comparison of Cas9 expression levels between the Cas9 individually-driven CRISPR/Cas9 system and mono-promoter-driven CRISPR/Cas9 system. (A) Schematics of the gRNA and Cas9 expression vectors tested in immunocytochemistry and RFLP analysis. (B) Representative images of HEK 293 cells stained with propidium iodide (DNA) and anti-Flag M2 monoclonal antibody (Cas9). HEK 293 cells (1.5x10^5) were transfected with one of the vectors shown in (A). Twenty four hours later, the cells were subjected to immunocytochemistry. (C) Representative images of western blot analysis. HEK 293 cells (1x10^5) were transfected with one of the vectors (1 µg) and vector sets (500 ng each) shown in (A), and the lysates of the cells were extracted 72 h after transfection. The cell lysates were subjected to Western blot analysis for Flag-M2 (Cas9) and ACTB expression. (D) Restriction fragment length polymorphism analysis. PCR products (300 ng) were subjected to agarose-gel electrophoresis after incubation with or without XcmI. Cleaved and uncleaved fragments indicate unmodified and modified genomes, respectively.

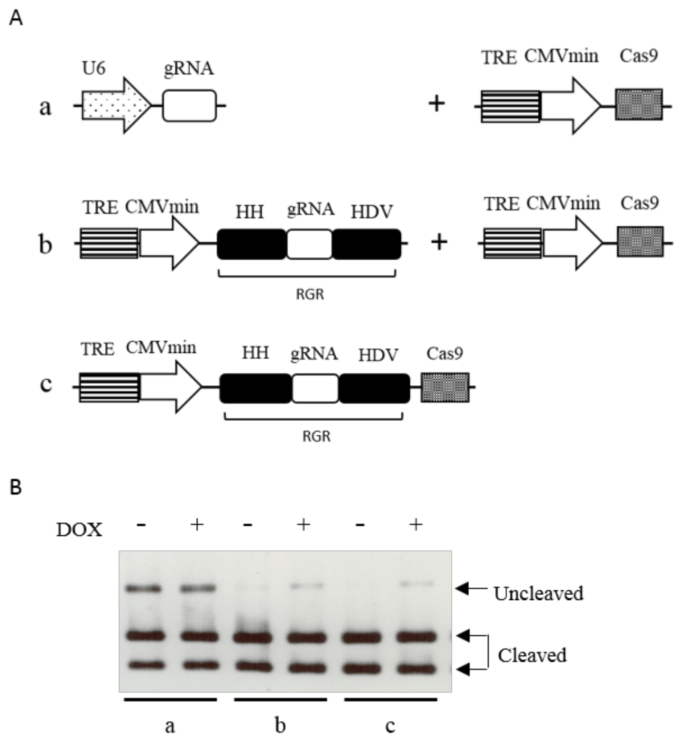

Supplementary Figure 6. Ubiquitous expression of Cas9 induce the off-doxycycline gene disruption. (A) Schematics of the gRNA and Cas9 expression vectors tested in doxycyclin-induced gene modification. All gRNAs targeted *HPRT1*. (B) The reverse tetracycline transactivator (rtTA) constitutively-expressing HEK 293 cells ($1x10^5$) were transfected with one of the vectors (1 μg) and vector sets (500 ng each) shown in (A). Twenty four hours later, the media were replaced to DMEM with 10% of tetracycline-free FBS, and incubated additional 48 hours with/without the doxycycline (1 μg/ml), then genomic DNAs of the cells were extracted 72 h after transfection. The DNA was subjected to PCR and the PCR products (300 ng) were subjected to agarose-gel electrophoresis after incubation with or without XcmI. Cleaved and uncleaved fragments indicate unmodified and modified genomes, respectively.

Supplementary Figure 7. Sequence of vectors and RGR.

>Sequence of pCAG-RGR-eGFP vector (from RGR to eGFP)

CAGGGCCTGATGAGTCCGTGAGGACGAAACGAGTAAGCTCGTCGCCCTGGCCGGTTCAGGCCCACGGTTT
TAGAGCTAGAAATAGCAAGTTAAAATAAGGCTAGTCCGTTATCAACTTGAAAAAGTGGCACCGAGTCGGT
GCTTTTGGCCGGCATGGTCCCAGCCTCCTCGCTGGCGCCGGCTGGGCAACATGCTTCGGCATGGCGAATG
GGACAATCACTAGTGAATTCACTCCTCAGGTGCAGGCTGCCTATCAGAAGGTGGTGGCTGGTGTGGCCAA
TGCCCTGGCTCACAAATACCACTGAGATCCACCATGGTGAGCAAGGGCGAGGAGCTGTTCACCGGGGTGG
TGCCCATCCTGGTCGAGCTGGACGGCGACGTAAACGGCCACAAGTTCAGCGTGTCCGGCGAGGGCGAGGG
CGATGCCACCTACGGCAAGCTGACCCTGAAGTTCATCTGCACCACCGGCAAGCTGCCCGTGCCCTGGCCC
ACCCTCGTGACCACCCTGACCTACGGCGTGCAGTGCTTCAGCCGCTACCCCGACCACATGAAGCAGCACG
ACTTCTTCAAGTCCGCCATGCCCGAAGGCTACGTCCAGGAGCGCACCATCTTCTTCAAGGACGACGGCAA
CTACAAGACCCGCGCCGAGGTGAAGTTCGAGGGCGACACCCTGGTGAACCGCATCGAGCTGAAGGGCATC
GACTTCAAGGAGGACGGCAACATCCTGGGGCACAAGCTGGAGTACAACTACAACAGCCACAACGTCTATA
TCATGGCCGACAAGCAGAAGAACGGCATCAAGGTGAACTTCAAGATCCGCCACAACATCGAGGACGGCAG
CGTGCAGCTCGCCGACCACTACCAGCAGAACACCCCCATCGGCGACGGCCCCGTGCTGCTGCCCGACAAC
CACTACCTGAGCACCCAGTCCGCCCTGAGCAAAGACCCCAACGAGAAGCGCGATCACATGGTCCTGCTGG
AGTTCGTGACCGCCGCCGGGATCACTCTCGGCATGGACGAGCTGTACAAGT

>Sequence of pCAG-RGR-IRES-eGFP vector (from RGR to eGFP)

CAGGGCCTGATGAGTCCGTGAGGACGAAACGAGTAAGCTCGTCGCCCTGGCCGGTTCAGGCCCACGGTTT
TAGAGCTAGAAATAGCAAGTTAAAATAAGGCTAGTCCGTTATCAACTTGAAAAAGTGGCACCGAGTCGGT
GCTTTTGGCCGGCATGGTCCCAGCCTCCTCGCTGGCGCCGGCTGGGCAACATGCTTCGGCATGGCGAATG
GGACAATCACTAGTGAATTCACTCCTCAGGTGCAGGCTGCCTATCAGAAGGTGGTGGCTGGTGTGGCCAA
TGCCCTGGCTCACAAATACCACTGAGATCCGCCCCTCTCCCTCCCCCCCCCCTAACGTTACTGGCCGAAG
CCGCTTGGAATAAGGCCGGTGTGCGTTTGTCTATATGTTATTTTCCACCATATTGCCGTCTTTTGGCAAT
GTGAGGGCCCGGAAACCTGGCCCTGTCTTCTTGACGAGCATTCCTAGGGGTCTTTCCCCTCTCGCCAAAG
GAATGCAAGGTCTGTTGAATGTCGTGAAGGAAGCAGTTCCTCTGGAAGCTTCTTGAAGACAAACAACGTC
TGTAGCGACCCTTTGCAGGCAGCGGAACCCCCCACCTGGCGACAGGTGCCTCTGCGGCCAAAAGCCACGT
GTATAAGATACACCTGCAAAGGCGGCACAACCCCAGTGCCACGTTGTGAGTTGGATAGTTGTGGAAAGAG
TCAAATGGCTCTCCTCAAGCGTATTCAACAAGGGGCTGAAGGATGCCCAGAAGGTACCCCATTGTATGGG
ATCTGATCTGGGGCCTCGGTACACATGCTTTACATGTGTTTAGTCGAGGTTAAAAAACGTCTAGGCCCC
CCGAACCACGGGGACGTGGTTTTCCTTTGAAAAACACGATGATAATATGGCCACAACCATGGTGAGCAAG
GGCGAGGAGCTGTTCACCGGGGTGGTGCCCATCCTGGTCGAGCTGGACGGCGACGTAAACGGCCACAAGT
TCAGCGTGTCCGGCGAGGGCGAGGGCGATGCCACCTACGGCAAGCTGACCCTGAAGTTCATCTGCACCAC
CGGCAAGCTGCCCGTGCCCTGGCCCACCCTCGTGACCACCCTGACCTACGGCGTGCAGTGCTTCAGCCGC
TACCCCGACCACATGAAGCAGCACGACTTCTTCAAGTCCGCCATGCCCGAAGGCTACGTCCAGGAGCGCA
CCATCTTCTTCAAGGACGACGGCAACTACAAGACCCGCGCCGAGGTGAAGTTCGAGGGCGACACCCTGGT
GAACCGCATCGAGCTGAAGGGCATCGACTTCAAGGAGGACGGCAACATCCTGGGGCACAAGCTGGAGTAC
AACTACAACAGCCACAACGTCTATATCATGGCCGACAAGCAGAAGAACGGCATCAAGGTGAACTTCAAGA
TCCGCCACAACATCGAGGACGGCAGCGTGCAGCTCGCCGACCACTACCAGCAGAACACCCCCATCGGCGA
CGGCCCCGTGCTGCTGCCCGACAACCACTACCTGAGCACCCAGTCCGCCCTGAGCAAAGACCCCAACGAG
AAGCGCGATCACATGGTCCTGCTGGAGTTCGTGACCGCCGCCGGGATCACTCTCGGCATGGACGAGCTGT
ACAAGTAA

>Sequence of pCAG-RGR-IRES-Cas9 vector (from RGR to Cas9)

CAGGGCCTGATGAGTCCGTGAGGACGAAACGAGTAAGCTCGTCGCCCTGGCCGGTTCAGGCCCACGGTTT
TAGAGCTAGAAATAGCAAGTTAAAATAAGGCTAGTCCGTTATCAACTTGAAAAAGTGGCACCGAGTCGGT
GCTTTTGGCCGGCATGGTCCCAGCCTCCTCGCTGGCGCCGGCTGGGCAACATGCTTCGGCATGGCGAATG
GGACAATCACTAGTGAATTCACTCCTCAGGTGCAGGCTGCCTATCAGAAGGTGGTGGCTGGTGTGGCCAA
TGCCCTGGCTCACAAATACCACTGAGATCCGCCCCTCTCCCTCCCCCCCCCCTAACGTTACTGGCCGAAG
CCGCTTGGAATAAGGCCGGTGTGCGTTTGTCTATATGTTATTTTCCACCATATTGCCGTCTTTTGGCAAT
GTGAGGGCCCGGAAACCTGGCCCTGTCTTCTTGACGAGCATTCCTAGGGGTCTTTCCCCTCTCGCCAAAG
GAATGCAAGGTCTGTTGAATGTCGTGAAGGAAGCAGTTCCTCTGGAAGCTTCTTGAAGACAAACAACGTC
TGTAGCGACCCTTTGCAGGCAGCGGAACCCCCCACCTGGCGACAGGTGCCTCTGCGGCCAAAAGCCACGT
GTATAAGATACACCTGCAAAGGCGGCACAACCCCAGTGCCACGTTGTGAGTTGGATAGTTGTGGAAAGAG
TCAAATGGCTCTCCTCAAGCGTATTCAACAAGGGGCTGAAGGATGCCCAGAAGGTACCCCATTGTATGGG
ATCTGATCTGGGGCCTCGGTACACATGCTTTACATGTGTTTAGTCGAGGTTAAAAAAACGTCTAGGCCCC
CCGAACCACGGGGACGTGGTTTTCCTTTGAAAAACACGATGATAATATGGCCACAACCATGGATTACAAG
GATGACGACGATAAGATCATGGCCCCAAAGAAGAAGCGGAAGGTCGGTATCCACGGAGTCCCAGCAGCCG
ACAAGAAGTACTCCATTGGGCTCGATATCGGCACAAACAGCGTCGGCTGGGCCGTCATTACGGACGAGTA
CAAGGTGCCGAGCAAAAAATTCAAAGTTCTGGGCAATACCGATCGCCACAGCATAAAGAAGAACCTCATT
GGCGCCCTCCTGTTCGACTCCGGGGAGACCGCCGAAGCCACGCGGCTCAAAAGAACAGCACGGCGCAGAT
ATACCCGCAGAAAGAATCGGATCTGCTACCTGCAGGAGATCTTTAGTAATGAGATGGCTAAGGTGGATGA
CTCTTTCTTCCATAGGCTGGAGGAGTCCTTTTTGGTGGAGGAGGATAAAAAGCACGAGCGCCACCCAATC
TTTGGCAATATCGTGGACGAGGTGGCGTACCATGAAAAGTACCCAACCATATATCATCTGAGGAAGAAGC
TTGTAGACAGTACTGATAAGGCTGACTTGCGGTTGATCTATCTCGCGCTGGCGCATATGATCAAATTTCG
GGGACACTTCCTCATCGAGGGGGACCTGAACCCAGACAACAGCGATGTCGACAAACTCTTTATCCAACTG
GTTCAGACTTACAATCAGCTTTTCGAAGAGAACCCGATCAACGCATCCGGAGTTGACGCCAAAGCAATCC
TGAGCGCTAGGCTGTCCAAATCCCGGCGGCTCGAAAACCTCATCGCACAGCTCCCTGGGGAGAAGAAGAA
CGGCCTGTTTGGTAATCTTATCGCCCTGTCACTCGGGCTGACCCCCAACTTTAAATCTAACTTCGACCTG
GCCGAAGATGCCAAGCTTCAACTGAGCAAAGACACCTACGATGATGATCTCGACAATCTGCTGGCCCAGA
TCGGCGACCAGTACGCAGACCTTTTTTTGGCGGCAAAGAACCTGTCAGACGCCATTCTGCTGAGTGATAT
TCTGCGAGTGAACACGGAGATCACCAAAGCTCCGCTGAGCGCTAGTATGATCAAGCGCTATGATGAGCAC
CACCAAGACTTGACTTTGCTGAAGGCCCTTGTCAGACAGCAACTGCCTGAGAAGTACAAGGAAATTTTCT
TCGATCAGTCTAAAAATGGCTACGCCGGATACATTGACGGCGGAGCAAGCCAGGAGGAATTTTACAAATT
TATTAAGCCCATCTTGGAAAAAATGGACGGCACCGAGGAGCTGCTGGTAAAGCTTAACAGAGAAGATCTG
TTGCGCAAACAGCGCACTTTCGACAATGGAAGCATCCCCCACCAGATTCACCTGGGCGAACTGCACGCTA
TCCTCAGGCGGCAAGAGGATTTCTACCCCTTTTTGAAAGATAACAGGGAAAAGATTGAGAAAATCCTCAC
ATTTCGGATACCCTACTATGTAGGCCCCCTCGCCCGGGGAAATTCCAGATTCGCGTGGATGACTCGCAAA
TCAGAAGAGACCATCACTCCCTGGAACTTCGAGGAAGTCGTGGATAAGGGGGCCTCTGCCCAGTCCTTCA
TCGAAAGGATGACTAACTTTGATAAAAATCTGCCTAACGAAAAGGTGCTTCCTAAACACTCTCTGCTGTA
CGAGTACTTCACAGTTTATAACGAGCTCACCAAGGTCAAATACGTCACAGAAGGGATGAGAAAGCCAGCA
TTCCTGTCTGGAGAGCAGAAGAAAGCTATCGTGGACCTCCTCTTCAAGACGAACCGGAAAGTTACCGTGA
AACAGCTCAAAGAAGACTATTTCAAAAAGATTGAATGTTTCGACTCTGTTGAAATCAGCGGAGTGGAGGA
TCGCTTCAACGCATCCCTGGGAACGTATCACGATCTCCTGAAAATCATTAAAGACAAGGACTTCCTGGAC
AATGAGGAGAACGAGGACATTCTTGAGGACATTGTCCTCACCCTTACGTTGTTTGAAGATAGGGAGATGA
TTGAAGAACGCTTGAAAACTTACGCTCATCTCTTCGACGACAAAGTCATGAAACAGCTCAAGAGGCGCCG
ATATACAGGATGGGGGCGGCTGTCAAGAAAACTGATCAATGGGATCCGAGACAAGCAGAGTGGAAAGACA
ATCCTGGATTTTCTTAAGTCCGATGGATTTGCCAACCGGAACTTCATGCAGTTGATCCATGATGACTCTC
TCACCTTTAAGGAGGACATCCAGAAAGCACAAGTTTCTGGCCAGGGGGACAGTCTTCACGAGCACATCGC
TAATCTTGCAGGTAGCCCAGCTATCAAAAAGGGAATACTGCAGACCGTTAAGGTCGTGGATGAACTCGTC
AAAGTAATGGGAAGGCATAAGCCCGAGAATATCGTTATCGAGATGGCCCGAGAGAACCAAACTACCCAGA
AGGGACAGAAGAACAGTAGGGAAAGGATGAAGAGGATTGAAGAGGGTATAAAAGAACTGGGGTCCCAAAT
CCTTAAGGAACACCCAGTTGAAAACACCCAGCTTCAGAATGAGAAGCTCTACCTGTACTACCTGCAGAAC
GGCAGGGACATGTACGTGGATCAGGAACTGGACATCAATCGGCTCTCCGACTACGACGTGGATCATATCG

TGCCCCAGTCTTTTCTCAAAGATGATTCTATTGATAATAAAGTGTTGACAAGATCCGATAAAAATAGAGG
GAAGAGTGATAACGTCCCCTCAGAAGAAGTTGTCAAGAAAATGAAAAATTATTGGCGGCAGCTGCTGAAC
GCCAAACTGATCACACAACGGAAGTTCGATAATCTGACTAAGGCTGAACGAGGTGGCCTGTCTGAGTTGG
ATAAAGCCGGCTTCATCAAAAGGCAGCTTGTTGAGACACGCCAGATCACCAAGCACGTGGCCCAAATTCT
CGATTCACGCATGAACACCAAGTACGATGAAAATGACAAACTGATTCGAGAGGTGAAAGTTATTACTCTG
AAGTCTAAGCTGGTCTCAGATTTCAGAAAGGACTTTCAGTTTTATAAGGTGAGAGAGATCAACAATTACC
ACCATGCGCATGATGCCTACCTGAATGCAGTGGTAGGCACTGCACTTATCAAAAAATATCCCAAGCTTGA
ATCTGAATTTGTTTACGGAGACTATAAAGTGTACGATGTTAGGAAAATGATCGCAAAGTCTGAGCAGGAA
ATAGGCAAGGCCACCGCTAAGTACTTCTTTTACAGCAATATTATGAATTTTTTCAAGACCGAGATTACAC
TGGCCAATGGAGAGATTCGGAAGCGACCACTTATCGAAACAAACGGAGAAACAGGAGAAATCGTGTGGGA
CAAGGGTAGGGATTTCGCGACAGTCCGGAAGGTCCTGTCCATGCCGCAGGTGAACATCGTTAAAAAGACC
GAAGTACAGACCGGAGGCTTCTCCAAGGAAAGTATCCTCCCGAAAAGGAACAGCGACAAGCTGATCGCAC
GCAAAAAAGATTGGGACCCCAAGAAATACGGCGGATTCGATTCTCCTACAGTCGCTTACAGTGTACTGGT
TGTGGCCAAAGTGGAGAAAGGGAAGTCTAAAAAACTCAAAAGCGTCAAGGAACTGCTGGGCATCACAATC
ATGGAGCGATCAAGCTTCGAAAAAAACCCCATCGACTTTCTCGAGGCGAAAGGATATAAAGAGGTCAAAA
AAGACCTCATCATTAAGCTTCCCAAGTACTCTCTCTTTGAGCTTGAAAACGGCCGGAAACGAATGCTCGC
TAGTGCGGGCGAGCTGCAGAAAGGTAACGAGCTGGCACTGCCCTCTAAATACGTTAATTTCTTGTATCTG
GCCAGCCACTATGAAAAGCTCAAAGGGTCTCCCGAAGATAATGAGCAGAAGCAGCTGTTCGTGGAACAAC
ACAAACACTACCTTGATGAGATCATCGAGCAAATAAGCGAATTCTCCAAAAGAGTGATCCTCGCCGACGC
TAACCTCGATAAGGTGCTTTCTGCTTACAATAAGCACAGGGATAAGCCCATCAGGGAGCAGGCAGAAAAC
ATTATCCACTTGTTTACTCTGACCAACTTGGGCGCGCCTGCAGCCTTCAAGTACTTCGACACCACCATAG
ACAGAAAGCGGTACACCTCTACAAAGGAGGTCCTGGACGCCACACTGATTCATCAGTCAATTACGGGGCT
CTATGAAACAAGAATCGACCTCTCTCAGCTCGGTGGAGACAGCAGGGCTGACCCCAAGAAGAAGAGGAAG
GTGTGA

>Sequence of universal vector (fromMCS to Cas9)

GTCGACGACGTCACCGGTGTACAGCTAGCGCGGCCGCGTATACCCGCGGGCATGCTCTAGAGCCTCTGCT
AACCATGTTCATGCCTTCTTCTTTTTCCTACAGCTCCTGGGCAACGTGCTGGTTATTGTGCTGTCTCATC
ATTTTGGCAAAGAATTCGATTCCGGAGAGACGGGATCCCGTCTCTGTTTTAGAGCTAGAAATAGCAAGTT
AAAATAAGGCTAGTCCGTTATCAACTTGAAAAAGTGGCACCGAGTCGGTGCTTTTGGCCGGCATGGTCCC
AGCCTCCTCGCTGGCGCCGGCTGGGCAACATGCTTCGGCATGGCGAATGGGACAATCACTAGTGAATTCA
CTCCTCAGGTGCAGGCTGCCTATCAGAAGGTGGTGGCTGGTGTGGCCAATGCCCTGGCTCACAAATACCA
CTGAGATCCGCCCCTCTCCCTCCCCCCCCCCTAACGTTACTGGCCGAAGCCGCTTGGAATAAGGCCGGTG
TGCGTTTGTCTATATGTTATTTTCCACCATATTGCCGTCTTTTGGCAATGTGAGGGCCCGGAAACCTGGC
CCTGTCTTCTTGACGAGCATTCCTAGGGGTCTTTCCCCTCTCGCCAAAGGAATGCAAGGTCTGTTGAATG
TCGTGAAGGAAGCAGTTCCTCTGGAAGCTTCTTGAAGACAAACAACGTCTGTAGCGACCCTTTGCAGGCA
GCGGAACCCCCCACCTGGCGACAGGTGCCTCTGCGGCCAAAAGCCACGTGTATAAGATACACCTGCAAAG
GCGGCACAACCCCAGTGCCACGTTGTGAGTTGGATAGTTGTGGAAAGAGTCAAATGGCTCTCCTCAAGCG
TATTCAACAAGGGGCTGAAGGATGCCCAGAAGGTACCCCATTGTATGGGATCTGATCTGGGGCCTCGGTA
CACATGCTTTACATGTGTTTAGTCGAGGTTAAAAAAACGTCTAGGCCCCCCGAACCACGGGGACGTGGTT
TTCCTTTGAAAAACACGATGATAATATGGCCACAACCATGGATTACAAGGATGACGACGATAAGATCATG
GCCCCAAAGAAGAAGCGGAAGGTCGGTATCCACGGAGTCCCAGCAGCCGACAAGAAGTACTCCATTGGGC
TCGATATCGGCACAAACAGCGTCGGCTGGGCCGTCATTACGGACGAGTACAAGGTGCCGAGCAAAAAATT
CAAAGTTCTGGGCAATACCGATCGCCACAGCATAAAGAAGAACCTCATTGGCGCCCTCCTGTTCGACTCC
GGGGAGACGGCCGAAGCCACGCGGCTCAAAAGAACAGCACGGCGCAGATATACCCGCAGAAAGAATCGGA
TCTGCTACCTGCAGGAGATCTTTAGTAATGAGATGGCTAAGGTGGATGACTCTTTCTTCCATAGGCTGGA
GGAGTCCTTTTTGGTGGAGGAGGATAAAAAGCACGAGCGCCACCCAATCTTTGGCAATATCGTGGACGAG
GTGGCGTACCATGAAAAGTACCCAACCATATATCATCTGAGGAAGAAGCTTGTAGACAGTACTGATAAGG

CTGACTTGCGGTTGATCTATCTCGCGCTGGCGCATATGATCAAATTTCGGGGACACTTCCTCATCGAGGG
GGACCTGAACCCAGACAACAGCGATGTCGACAAACTCTTTATCCAACTGGTTCAGACTTACAATCAGCTT
TTCGAAGAGAACCCGATCAACGCATCCGGAGTTGACGCCAAAGCAATCCTGAGCGCTAGGCTGTCCAAAT
CCCGGCGGCTCGAAAACCTCATCGCACAGCTCCCTGGGGAGAAGAAGAACGGCCTGTTTGGTAATCTTAT
CGCCCTGTCACTCGGGCTGACCCCCAACTTTAAATCTAACTTCGACCTGGCCGAAGATGCCAAGCTTCAA
CTGAGCAAAGACACCTACGATGATGATCTCGACAATCTGCTGGCCCAGATCGGCGACCAGTACGCAGACC
TTTTTTTGGCGGCAAAGAACCTGTCAGACGCCATTCTGCTGAGTGATATTCTGCGAGTGAACACGGAGAT
CACCAAAGCTCCGCTGAGCGCTAGTATGATCAAGCGCTATGATGAGCACCACCAAGACTTGACTTTGCTG
AAGGCCCTTGTCAGACAGCAACTGCCTGAGAAGTACAAGGAAATTTTCTTCGATCAGTCTAAAAATGGCT
ACGCCGGATACATTGACGGCGGAGCAAGCCAGGAGGAATTTTACAAATTTATTAAGCCCATCTTGGAAAA
AATGGACGGCACCGAGGAGCTGCTGGTAAAGCTTAACAGAGAAGATCTGTTGCGCAAACAGCGCACTTTC
GACAATGGAAGCATCCCCCACCAGATTCACCTGGGCGAACTGCACGCTATCCTCAGGCGGCAAGAGGATT
TCTACCCCTTTTTGAAAGATAACAGGGAAAAGATTGAGAAAATCCTCACATTTCGGATACCCTACTATGT
AGGCCCCCTCGCCCGGGGAAATTCCAGATTCGCGTGGATGACTCGCAAATCAGAAGAGACCATCACTCCC
TGGAACTTCGAGGAAGTCGTGGATAAGGGGGCCTCTGCCCAGTCCTTCATCGAAAGGATGACTAACTTTG
ATAAAAATCTGCCTAACGAAAAGGTGCTTCCTAAACACTCTCTGCTGTACGAGTACTTCACAGTTTATAA
CGAGCTCACCAAGGTCAAATACGTCACAGAAGGGATGAGAAAGCCAGCATTCCTGTCTGGAGAGCAGAAG
AAAGCTATCGTGGACCTCCTCTTCAAGACGAACCGGAAAGTTACCGTGAAACAGCTCAAAGAAGACTATT
TCAAAAAGATTGAATGTTTCGACTCTGTTGAAATCAGCGGAGTGGAGGATCGCTTCAACGCATCCCTGGG
AACGTATCACGATCTCCTGAAAATCATTAAAGACAAGGACTTCCTGGACAATGAGGAGAACGAGGACATT
CTTGAGGACATTGTCCTCACCCTTACGTTGTTTGAAGATAGGGAGATGATTGAAGAACGCTTGAAAACTT
ACGCTCATCTCTTCGACGACAAAGTCATGAAACAGCTCAAGAGGCGCCGATATACAGGATGGGGGCGGCT
GTCAAGAAAACTGATCAATGGGATCCGAGACAAGCAGAGTGGAAAGACAATCCTGGATTTTCTTAAGTCC
GATGGATTTGCCAACCGGAACTTCATGCAGTTGATCCATGATGACTCTCTCACCTTTAAGGAGGACATCC
AGAAAGCACAAGTTTCTGGCCAGGGGGACAGTCTTCACGAGCACATCGCTAATCTTGCAGGTAGCCCAGC
TATCAAAAAGGGAATACTGCAGACCGTTAAGGTCGTGGATGAACTCGTCAAAGTAATGGGAAGGCATAAG
CCCGAGAATATCGTTATCGAGATGGCCCGAGAGAACCAAACTACCCAGAAGGGACAGAAGAACAGTAGGG
AAAGGATGAAGAGGATTGAAGAGGGTATAAAAGAACTGGGGTCCCAAATCCTTAAGGAACACCCAGTTGA
AAACACCCAGCTTCAGAATGAGAAGCTCTACCTGTACTACCTGCAGAACGGCAGGGACATGTACGTGGAT
CAGGAACTGGACATCAATCGGCTCTCCGACTACGACGTGGATCATATCGTGCCCCAGTCTTTTCTCAAAG
ATGATTCTATTGATAATAAAGTGTTGACAAGATCCGATAAAAATAGAGGGAAGAGTGATAACGTCCCCTC
AGAAGAAGTTGTCAAGAAAATGAAAAATTATTGGCGGCAGCTGCTGAACGCCAAACTGATCACACAACGG
AAGTTCGATAATCTGACTAAGGCTGAACGAGGTGGCCTGTCTGAGTTGGATAAAGCCGGCTTCATCAAAA
GGCAGCTTGTTGAGACACGCCAGATCACCAAGCACGTGGCCCAAATTCTCGATTCACGCATGAACACCAA
GTACGATGAAAATGACAAACTGATTCGAGAGGTGAAAGTTATTACTCTGAAGTCTAAGCTGGTCTCAGAT
TTCAGAAAGGACTTTCAGTTTTATAAGGTGAGAGAGATCAACAATTACCACCATGCGCATGATGCCTACC
TGAATGCAGTGGTAGGCACTGCACTTATCAAAAAATATCCCAAGCTTGAATCTGAATTTGTTTACGGAGA
CTATAAAGTGTACGATGTTAGGAAAATGATCGCAAAGTCTGAGCAGGAAATAGGCAAGGCCACCGCTAAG
TACTTCTTTTACAGCAATATTATGAATTTTTTCAAGACCGAGATTACACTGGCCAATGGAGAGATTCGGA
AGCGACCACTTATCGAAACAAACGGAGAAACAGGAGAAATCGTGTGGGACAAGGGTAGGGATTTCGCGAC
AGTCCGGAAGGTCCTGTCCATGCCGCAGGTGAACATCGTTAAAAAGACCGAAGTACAGACCGGAGGCTTC
TCCAAGGAAAGTATCCTCCCGAAAAGGAACAGCGACAAGCTGATCGCACGCAAAAAAGATTGGGACCCCA
AGAAATACGGCGGATTCGATTCTCCTACAGTCGCTTACAGTGTACTGGTTGTGGCCAAAGTGGAGAAAGG
GAAGTCTAAAAAACTCAAAAGCGTCAAGGAACTGCTGGGCATCACAATCATGGAGCGATCAAGCTTCGAA
AAAAACCCCATCGACTTTCTCGAGGCGAAAGGATATAAAGAGGTCAAAAAAGACCTCATCATTAAGCTTC
CCAAGTACTCTCTCTTTGAGCTTGAAAACGGCCGGAAACGAATGCTCGCTAGTGCGGGCGAGCTGCAGAA
AGGTAACGAGCTGGCACTGCCCTCTAAATACGTTAATTTCTTGTATCTGGCCAGCCACTATGAAAAGCTC
AAAGGGTCTCCCGAAGATAATGAGCAGAAGCAGCTGTTCGTGGAACAACACAAACACTACCTTGATGAGA
TCATCGAGCAAATAAGCGAATTCTCCAAAAGAGTGATCCTCGCCGACGCTAACCTCGATAAGGTGCTTTC
TGCTTACAATAAGCACAGGGATAAGCCCATCAGGGAGCAGGCAGAAAACATTATCCACTTGTTTACTCTG

ACCAACTTGGGCGCGCCTGCAGCCTTCAAGTACTTCGACACCACCATAGACAGAAAGCGGTACACCTCTA
CAAAGGAGGTCCTGGACGCCACACTGATTCATCAGTCAATTACGGGGCTCTATGAAACAAGAATCGACCT
CTCTCAGCTCGGTGGAGACAGCAGGGCTGACCCCAAGAAGAAGAGGAAGGTGTGA

>HPRT1 gRNA (Target site recognition sequence to gRNA scafold)
CCCTGGCCGGTTCAGGCCCACGGTTTTAGAGCTAGAAATAGCAAGTTAAAATAAGGCTAGTCCGTTATCA
ACTTGAAAAAGTGGCACCGAGTCGGTGCTTTTT

>HPRT1 RGR (HH ribozyme to HDV ribozyme)
CAGGGCCTGATGAGTCCGTGAGGACGAAACGAGTAAGCTCGTCGCCCTGGCCGGTTCAGGCCCACGGTTT
TAGAGCTAGAAATAGCAAGTTAAAATAAGGCTAGTCCGTTATCAACTTGAAAAAGTGGCACCGAGTCGGT
GCTTTTGGCCGGCATGGTCCCAGCCTCCTCGCTGGCGCCGGCTGGGCAACATGCTTCGGCATGGCGAATG
GGAC

>FAN1 RGR (HH ribozyme to HDV ribozyme)
GAAGTCCTGATGAGTCCGTGAGGACGAAACGAGTAAGCTCGTCGACTTCGTTCAAGTGGATCCGTTTTAG
AGCTAGAAATAGCAAGTTAAAATAAGGCTAGTCCGTTATCAACTTGAAAAAGTGGCACCGAGTCGGTGCT
TTTGGCCGGCATGGTCCCAGCCTCCTCGCTGGCGCCGGCTGGGCAACATGCTTCGGCATGGCGAATGGGA
C

Target locus	Target sequence	Primer sequence for RGR
HPRT1	5’-GCCCTGGCCGGTTCAGGCCCACG**CGG**	Fw: 5’-CAGGGCCTGATGAGTCCGTGAGGACGAAACGAGTAAGCTCGTCGCCCTGGCCGGTTCAGGC<br>CCACGGTTTTAGAGCTAGAAATAGC
		Rv: 5’-GTCCCATTCGCCATGCCGAAGCATGTTGCCCAGCCGGCGCCAGCGAGGAGGCTGGGACCATGCCGGCCAAAAG<br>CACCGACTCGGTGCCACTTTTTCAAGTTGATAACGGACTAGCCTTATTTTAACTTGCTATTTCTAGCTCTAAAAC
FAN1	5’-GACTTCGTTCAAGTGGATCC**AGG**	Fw: 5’-GAAGTCCTGATGAGTCCGTGAGGACGAAACGAGTAAGCTCGTCGACTTCGTTCAAGTGGATCC<br>GTTTTAGAGCTAGAAATAGC
		Rv: 5’-CGACTAGTGTCCCATTCGCCATGCCGAAGCATGTTGCCCAGCCGGCGCCAGCGAGGAGGCTGGGACCATGCCGGC<br>CAAAAGCACCGACTCGGTGCCACTTTTTCAAGTTGATAACGGACTAGCCTTATTTTAACTTGCTATTTCTAGCTC

Supplementary Table 1. Target sequence of human *HPRT1* and *FAN1* locus tested in this study and primers for RGR. Bold characters show PAM domain.

Multi cloning site	Fw: 5’−TCGACGACGTCACCGGTGTACAGCTAGCGCGGCCGCGTATACCCGCGGGCATGCT
	Rv: 5’−CTAGAGCATGCCCGCGGGTATACGCGGCCGCGCTAGCTGTACACCGGTGACGTCG
RGR	Fw: 5’−CCGGAGAGACGGGATCCCGTCTCTGTTTTAGAGCTAGAAATAGC
	Rv: 5’−GTCCCATTCGCCATGCCGAAGCATGTTGCCCAGCCGGCGCCAGCGAGGAGGCTGGGACCATGCCGGCCAAAAG<br>CACCGACTCGGTGCCACTTTTTCAAGTTGATAACGGACTAGCCTTATTTTAACTTGCTATTTCTAGCTCTAAAAC

Supplementary Table 2. Primer sequence for universal vector.

Target locus	Primer sequence
HPRT1	Fw: 5’-GAGAAAATTCCCACGGCTACCTAG
	Rv: 5’-CAGAGCTGTAGTGGGGCTTC
FAN1	Fw: 5’-TCCCACTTTTGGTGATTTCAAGTCAAG
	Rv: 5’-CTCTGTGGACTAGAACCGGC
Off-target1	Fw: 5’-AGGACTGAGTCGGCTCCTAA
	Rv: 5’-GCACAAGGGGGCCTTTGTTC
Off-target2	Fw: 5’-GAGTGGTGAGGTGTTGGGAG
	Rv: 5’-GAGGCCCTTCTTCCTGAACG

Supplementary Table 3. Primer sequence for RFLP analysis and T7E1 assay.

RGR	Fw: 5’-GAGTCCGTGAGGACGAAACG
	Rv: 5’-CGCCATGCCGAAGCATGTTG
GAPDH	Fw: 5’-CATCACCATCTTCCAGGAGC
	Rv: 5’-GCAGGGATGATGTTCTGGAG

Supplementary Table 4. Primer sequence for RT-PCR

Supplementary References

1. Fujii, W., Kawasaki, K., Sugiura, K. & Naito, K. Efficient generation of large-scale genome-modified mice using gRNA and CAS9 endonuclease. *Nucleic Acids Res* **41**, e187 (2013).
2. Fujii, W., Onuma, A., Sugiura, K. & Naito, K. Efficient generation of genome-modified mice via offset-nicking by CRISPR/Cas system. *Biochem Biophys Res Commun* **445**, 791-794 (2014).
3. Laemmli, U. K. Cleavage of structural proteins during the assembly of the head of bacteriophage T4. *Nature* **227**, 680-685 (1970).
4. Tomita, K., Ogawa, T., Uozumi, T., Watanabe, K. & Masaki, H. A cytotoxic ribonuclease which specifically cleaves four isoaccepting arginine tRNAs at their anticodon loops. *Proc Natl Acad Sci U S A* **97**, 8278-8283 (2000).
5. Ogawa, T., Shimizu, A., Takahashi, K., Hidaka, M. & Masaki, H. Mitochondrial tRNA cleavage by tRNA-targeting ribonuclease causes mitochondrial dysfunction observed in mitochondrial disease. *Biochem Biophys Res Commun* **451**, 131-136 (2014).
